# Salinity and Time Can Alter Epibacterial Communities of an Invasive Seaweed

**DOI:** 10.3389/fmicb.2019.02870

**Published:** 2020-01-15

**Authors:** Mahasweta Saha, Robert M. W. Ferguson, Shawn Dove, Sven Künzel, Rafael Meichssner, Sven C. Neulinger, Finn Ole Petersen, Florian Weinberger

**Affiliations:** ^1^Benthic Ecology, GEOMAR Helmholtz Centre for Ocean Research, Kiel, Germany; ^2^The School of Life Sciences, University of Essex, Colchester, United Kingdom; ^3^Marine Ecology and Biodiversity, Plymouth Marine Laboratory, Plymouth, United Kingdom; ^4^Centre for Biodiversity and Environment Research, University College London, London, United Kingdom; ^5^Institute of Zoology, Zoological Society of London, London, United Kingdom; ^6^Max Planck Institute for Evolutionary Biology, Plön, Germany; ^7^Department of Biology, Botanical Institute, Christian-Albrechts-University, Kiel, Germany; ^8^Coastal Research & Management, Kiel, Germany; ^9^omics2view.consulting GbR, Kiel, Germany; ^10^Department of Biology, Institute for General Microbiology, Christian-Albrechts-University, Kiel, Germany

**Keywords:** salinity, time, invasive, seaweed, epibacteria, community, metagenome

## Abstract

The establishment of epibacterial communities is fundamental to seaweed health and fitness, in modulating ecological interactions and may also facilitate adaptation to new environments. Abiotic factors like salinity can determine bacterial abundance, growth and community composition. However, influence of salinity as a driver of epibacterial community composition (until species level) has not been investigated for seaweeds and especially under long time scales. We also do not know how abiotic stressors may influence the ‘core’ bacterial species of seaweeds. Following an initial (immediately after field collection) sampling of epibacterial community of an invasive red seaweed *Agarophyton vermicullophylum*, we conducted a long term mesocosm experiment for 5 months, to examine the influence of three different salinities (low, medium and high) at two different time points (3 months after start of experiment and 5 months, i.e., at the end of experiment) on the epibacterial community richness and composition of *Agarophyton*. Metagenomic sequencing showed that epibacterial communities changed significantly according to salinity and time points sampled. Epibacterial richness was significantly different between low and high salinities at both time points. Epibacterial richness also varied significantly between 3 months (after start of experiment) and 5 months (end of experiment) within low, medium and high salinity level. Irrespective of salinity levels and time points sampled 727 taxa consistently appeared in all *Agarophyton* samples hinting at the presence of core bacterial species on the surface of the alga. Our results indicate that both salinity and time can be major driving forces in structuring epibacterial communities of seaweeds with respect to richness and β-diversity. We highlight the necessity of conducting long term experiments allowing us to detect and understand epibacterial succession over time on seaweeds.

## Introduction

Salinity can be an overall driver of ecosystem function (Smyth and Elliott, 2016) and is considered as one of the most influential environmental determinants, not only for distribution of benthic and pelagic organisms (Palmer et al., 2011; Darr et al., 2014) but also for microbial community composition (Lozupone and Knight, 2007). Salinity can change community structure and ecological function in Archaea (Xie et al., 2014) and affect bacterial abundance, growth and activity (Caporaso et al., 2011). Salinity fluctuations and their subsequent effect on aquatic organisms are more noticeable in estuaries and brackish water ecosystems as these habitats are characterized by a more or less pronounced salinity gradient (Telesh et al., 2013). The Baltic Sea is one of the world’s largest semi-enclosed brackish water seas with prolonged periods of high or low water caused mainly by atmospheric fluctuations. Presence of strong estuarine-like salinity gradients can determine geographical distribution of most species in the Baltic Sea (Smyth and Elliott, 2016). In the Northern Baltic where salinities are around 1–3 psu, only 1–3 groups of macrozoobenthos are found which are poor in functional complexity compared to 8–20 complex groups in the Southern Baltic where salinities are around 25–30 psu (Bonsdorff and Pearson, 1999). The salinity gradient in the Baltic is also an important factor structuring epibacterial community composition and/or diversity on the native brown seaweed *Fucus vesiculosus* (Stratil et al., 2014). However, salinity is expected to decrease over the coming decades in the Baltic Sea (BACC Author Team, 2008; BACC II Author Team, 2015) and is considered as a regional climate change induced stressor.

Salinity is considered one of the most significant factors limiting distribution of species, including seaweeds in aquatic environments (Ojaveer et al., 2010). The surface of seaweeds is the ecological and functional interface between the host and the environment (Wahl, 2008). Seaweeds are host to a diverse array of bacteria colonizing their surfaces – together forming a single entity called the seaweed holobiont (Egan et al., 2013). Some of these bacteria are essential for the health and normal morphological development of the seaweed host (Wichard et al., 2015; Saha and Weinberger, 2019), consumption of organic matter and nitrogen source (De Oliveira et al., 2012) and provide associational chemical defense against micro and macro colonizers (reviewed by Dahms and Dobretsov, 2017; Saha et al., 2017; Saha and Weinberger, 2019). Given this crucial ecological role of epibiotic bacteria, it is important to know how composition and richness of these epibacteria is controlled by the host and environmental factors. Role of the seaweed holobiont surface chemistry in manipulating epibacterial abundance (reviewed by da Gama et al., 2014; Saha et al., 2017) and composition (Lachnit et al., 2010; Longford et al., 2019) has been documented to some extent. A recent study on the Mediterranean brown algae *Taonia atomaria* has demonstrated that seasonal variations in surface metabolites of the alga which are known to function as antifouling metabolites in brown seaweeds (Saha et al., 2012) was positively correlated to the change in epibacterial community composition of the alga (Paix et al., 2019). Recent studies have also demonstrated that epibacterial communities on seaweeds can be altered under environmental stressors like high temperature (Mensch et al., 2016; Minich et al., 2018). However, to our knowledge, impact of salinity on epibacterial communities of seaweeds has been rarely investigated except by Stratil et al. (2014) on the NE Atlantic native brown seaweed *Fucus vesiculosus*.

DGGE-based studies detected evidence that specific sets of bacterial core taxa are consistently associated with different species of macroalgae (Tujula et al., 2010; Lachnit et al., 2011). Few examples suggest that seaweed hosts harbor ‘core’ endosymbiotic microorganisms, thereby protecting them to some degree from environmental impact (Hollants et al., 2013). Although epibacteria can determine the health and fitness of the algal host, we hardly know yet whether seaweeds have a ‘core’ microbiome (except study by Lachnit et al., 2009; Tujula et al., 2010; Lachnit et al., 2011) and if they can maintain the core microbiome in response to abiotic stressors and over time.

*Agarophyton vermiculophyllum* (Ohmi) (Gurgel et al., 2018) [Synonym: *Gracilaria vermiculophyllum* (Ohmi) Papenfuss, hereafter: *Agarophyton*] is a red seaweed native to the Northwest Pacific region (Ohmi, 1956; Yoshida, 1998). During the last two decades, *Agarophyton* has successfully invaded many coastal ranges in the East Pacific, West Atlantic, East Atlantic, and Mediterranean Sea (Thomsen et al., 2013). This alga tends to cause changes in the flora and fauna in its invaded range and is considered as a potent invader in Europe (Nyberg, 2007). Invasive populations of this alga are of lower palatability than native populations to both native and non-native generalist herbivores (Hammann et al., 2013) and more resistant to native and non-native epibionts (Wang et al., 2017). These invasive populations also exhibited an adaptation of chemical defense toward bacterial colonizers from the invaded region (Saha et al., 2016). Additionally, *Agarophyton* has a remarkable tolerance towards low salinity (Nyberg, 2007; Thomsen et al., 2007) and it mainly grows in estuaries and brackish water lagoons (Freshwater et al., 2006).

Using the model seaweed *Agarophyton*, in the current study we conducted a 5 months mesocosm experiment to investigate the impact of three different salinities on the epibacterial abundance, richness and community associated with *Agarophyton* for three different time points by metagenome sequencing. Specifically, we asked if the composition and richness of epibiotic biofilms differed (i) with salinity (ii) at different time points and whether *Agarophyton* can maintain a set of ‘core’ bacterial taxa irrespective of different salinity conditions and time points of sampling (during the salinity treatment).

## Materials and Methods

### Collection of *Agarophyton vermiculophyllum*

In May 2015, individuals of the invasive red alga *Agarophyton vermiculophyllum* (Ohmi) were collected from Nordstrand, Germany (54°29.166′N, 8°48′.746′E) and brought to the laboratory in a cooler box within 2 h after collection. They were maintained in 20 L aquaria for 16 h at a salinity of 33 psu (approximate salinity value at the collection site) at 15°C under constant aeration and a photon flux density of 75 μmol m^2^ s^1^ (12 h of light per d) until commencement of the salinity treatment.

### Overview of Experimental Setup

The salinity experiment was conducted for 5 months between May – October 2015 in a climate chamber at 15°C. Light intensity was 75 μmol m^2^ s^1^ (12 h of light per d) throughout the duration of the experiment. The setups consisted of 3 salinity levels and were classified as: low = 8.5 psu (± 0.51 SD), medium = 16.5 psu (±1.44 SD) and high = 25.5 psu (±1.44 SD). These levels are within the distributional range of *Agarophyton* in the Baltic (Bonsdorff and Pearson, 1999). To ensure equal supply of the natural pool of microbial colonizers to all salinity levels, low salinity water made up from natural sea water served as the stock solution for the preparation of medium and high salinity water. The low salinity level was obtained by diluting freshly collected Kiel Fjord water with fresh water (0 psu). Salinity in the Kiel Fjord during the entire experiment was about 16.5 psu (±0.5 SD) and was measured weekly. Low salinity water was prepared by diluting the Kiel Fjord water by 50%. Medium and high salinity was prepared by adding salt (Instant Ocean, Blacksburg, VA, United States) to the low salinity water. Water for each salinity level was prepared 48h in advance before being distributed to the tanks.

The algal individuals were distributed between the three salinity levels. Each salinity level had five replicated individuals in separate plastic aquaria of 8 L volume. Each aquarium received 6 L of water of the respective salinity. Water was exchanged manually in all the aquaria three times a week.

### Quantification of Epibacterial Cells on *Agarophyton vermiculophyllum*

In order to assess the effect of salinities and time points on bacterial cell density on surface of the alga, epibacterial cell density from T1 and T2 (*n* = 5 for each salinity level and each time point) was determined via fluorescence microscopy of DAPI-stained bacterial cells. Epibacterial cell density samples from T0 were generated from 5 individuals prior to start of the treatment to obtain cell density values from T0. For methodological details, see Supplementary Appendix S1 in supplementary information. Briefly, the bacteria attached to the surface of a defined quantity of *Agarophyton* were suspended in sterile filtered (0.20 μm) seawater (SSW) via sonification and vortexing with sterile glass beads. The cells were then enriched on nitrocellulose filters, stained using standard techniques, and counted under epifluorescence microscopy, and the results were standardized based on the sampled thallus area.

### Sampling of Epibacterial Community From *Agarophyton vermiculophyllum*

Epibacterial community samples from *Agarophyton* were generated at three time points [May (T0), August (T1) and October 2015 (T2)]. Epibacterial community samples from T0 were generated from 5 individuals prior to the start of the treatment, i.e., 16 h after field collection to obtain community composition, richness and diversity data at T0. A defined quantity (ca. 1 g fresh weight) of algal individuals was rinsed with SSW to remove loosely attached particles. The bacteria attached to the surface of ca. 1 g of *Agarophyton* were suspended in 30 ml SSW via sonification (30 s) and vortexing for 3 min with sterile glass beads. The suspended material was pre-filtered using sterile 5 μm filters to eliminate microalgal DNA and cyanobacteria (>5 μm). Cells from the filtrate were then enriched on nitrocellulose filters. The filters were then stored individually in sterile Eppendorf tubes at −80°C until DNA extraction. Replication level was 5 for low, medium and high salinity level in T1 and T2. However, only two algal replicates could be harvested from high salinity in T2, as much of the other three replicates were fragmented, severely degraded and not enough fresh algal (ca. 1 g) material was left for extracting DNA in sufficient quantities. Exclusion of three degraded samples were done to avoid contamination of endophytic bacteria from fragmented and decayed individuals and thereby misinterpretation of results. For T0 samples, salinity of the SSW (artificial) was 25.5 psu. However, for sampling at time points T1 and T2, *Agarophyton* originating from low, medium and high treatment was rinsed with artificial SSW of respective salinities, i.e., 8.5, 16.5, and 25.5 psu to avoid osmotic stress to the bacterial cells.

### Sampling of Epibacterial Community From Tank Walls

The walls of the aquaria were exposed to the same colonizer pool as *Agarophyton* and served used as non-living control substrata. Epibacterial community were sampled from them only at the end of the experiment in October (T2). For this purpose, biofilm attached to 1 cm^2^ of the tank wall was scraped out using a sterile scalpel and suspended in 30 ml artificial SSW by means of sonification (30 s) and vortexing for 3 min with sterile glass beads. Filters containing epibacteria were prepared as described above. Replication level was four in low, three in medium salinity level and one in high salinity level. This was due to loss of one replicate sample (out of 2: only two were sampled from tank walls as only two algae samples from high salinity at T2 could be harvested for community composition work) from the high salinity treatment. Due to low numbers of sequences from one sample in low salinity and two samples from medium level, these samples were removed during sample normalization (see statistical analysis).

### DNA Extraction and Metagenomic Sequencing

DNA was extracted from the filters (generated above) with the MoBio PowerWater DNA Isolation Kit (Dianova GmbH, Hamburg, Germany) following the manufacturer’s protocol for nitrocellulose filters. Given that community composition is tissue dependent in seaweeds, to ensure consistency, algal samples were always taken from the growing tip of the alga. DNA was eluted with nuclease-free water and stored at −80°C until further analysis.

For the sample preparation we used the Nextera XT DNA library preparation kit (FC-131-1024) from Illumina. The DNA was tagmented, amplified by PCR (12 cycles) and purified by AMPure XP (Beckman and Coulter). At the end, the individual samples were pooled equimolar and placed on a NextSeq 500 (Illumina) and sequenced with the NextSeq Mid-Output v2 Kit (300 cycles) (Illumina, FC-404-2003).

### Sequence Quality Filtering and *de novo* Contiguous Sequence Assembly

In total 1,606,698,064 reads were obtained, of which 1,236,235,985 (77%) passed the quality filter described below, giving 308,968,716 contiguous sequences (hereafter referred to as contigs). Reads were trimmed when the average Phred quality score within a 5 base pair (bp) sliding window dropped below 30, using Trimmomatic v0.36 (Bolger et al., 2014). Reads were removed if they were smaller than 20 bp, contained ambiguous bases, or were of low complexity (e.g., >8 bp homopolymers, di-/trinucleotide repeats). For *de novo* contig assembly, separate pre-assemblies of the filtered reads of individual samples were constructed with MEGAHIT v1.1.1 with the preset ‘meta-large’ (Li et al., 2015, 2016). Pre-assembled contigs ≥2000 bp were merged and de-replicated with the program Dedupe v36.30 from the BBTools suite (Bushnell, 2016). The merged contigs were then scaffolded with SSPACE v3.0, using the default parameters (Boetzer et al., 2010). The filtered reads were back-mapped onto the scaffolded assembly with BBMap v36.30 (Bushnell, 2016), using a K-mer length of 12. The resulting alignments were filtered with SAMtools v1.3.1 (Li, 2009) to retain only unambiguously mapped reads with a minimum MAPQ alignment quality score of 20. Here, only contigs with a minimum coverage of 10 were considered reliable and retained.

### Taxonomic Annotation of Contigs

Taxonomic annotation of contigs was accomplished with Kraken v0.10.6 (Wood and Salzberg, 2014). A reference database for assignment was constructed from the NCBI Reference Sequence Database downloaded on the 16/12/2016 (Pruitt et al., 2007). Low-complexity regions within the reference sequences were masked using DustMasker v1.0.0 (Morgulis et al., 2006) from the BLAST + v2.5.0 package (Altschul et al., 1990; Camacho et al., 2009) with the default parameters. Filtered contigs were annotated against a separate database for bacteria, archaea, viruses, protozoa, and fungi. Unique assignments for bacteria were retained. Contigs that matched to more than one database were reported as undetermined. In total 120697 contigs could be assigned to 2538 different bacterial taxa.

### Statistical Analysis

Statistical analysis was carried out in R (R Development Core Team, 2015) and the cited associated packages. A Generalized Linear Model (GLM) was used to model the epibacterial count data from DAPI staining with respect to time and salinity. Model checking deemed a negative binomial fit the most appropriate, in particular for dealing with over dispersion. Stepwise regression was used to select the minimum adequate model with backward elimination from the maximal model. For all tests, an alpha value of *p* < 0.05 was used.

Analysis of the sequencing data was carried out using the R package Vegan (Oksanen et al., 2015) as described in Dumbrell et al. (2017). Read depth was normalized across the samples by rarefying to 16,484 reads. Because of this, three samples were excluded due to low sequence depth (one sample at T0 and two medium salinity samples from tank walls at T2). One replicate from T0 and one replicate from low salinity at T1 could not be included due to poor yield of sequences after quality control. As contigs could have come from the same bacterial genome, taxa richness was calculated based on the number of unique taxa detected at the species level. Significance between taxa richness was evaluated using a Kruskal–Wallis test (Kruskal and Wallis, 1952), for *post hoc* analysis Dunn’s test for multiple comparisons with rank sums was used and *p*-values were adjusted with the Benjamini–Hochberg method (Dunn, 1964). To evaluate changes in bacterial community composition (beta diversity), a distance matrix using the Jaccard index was calculated based on presence/absence of bacterial taxa and visualized with non-metric multidimensional scaling (NMDS). To test for differences between treatments, permutation-based multivariate analysis of variance (PERMANOVA) on the distance matrix was carried out with 1000 randomizations (Anderson and Walsh, 2013).

To identify taxa that were significantly differently abundant between treatments, pair wise comparisons of abundance of the contigs were carried out using DESeq2 using the Wald test (Love et al., 2014). Dispersions to the mean were fitted with a local regression of log dispersions over log base mean, and *p*-values were adjusted with the Benjamini–Hochberg method (Love et al., 2014). The un-normalized count data for each contig was used (as the analysis internally normalizes the library size), but data was pre-filtered to remove rare contigs (top 200) or any contigs with zero counts to keep the results manageable. The core epibacterial microbiota was defined as taxa that were present in all replicates for all salinity levels (without normalization). Relative abundance for taxa was combined for contigs that were identified and assigned to the same species for this analysis.

## Results

### Effect of Salinity and Time on Epibacterial Abundance

**FIGURE 1:**
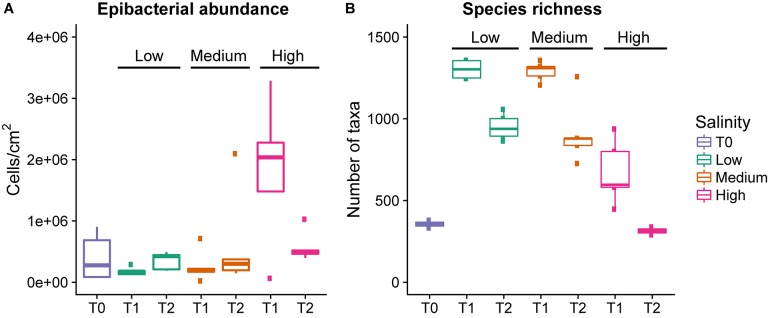
Epibacterial abundance **(A)** and species richness **(B)** at time points T0, T1, and T2 for each salinity level. Epibacterial abundance on algal surface is based on DAPI counts and expressed as cells/cm2 of alga (*n* = 5). Species richness is the number of distinct taxa at the species level observed at time points T0, T1, and T2 for each salinity level [*n* = 5, except T0 (*n* = 3) and T2 high salinity (*n* = 2), and T1 low salinity (*n* = 4)]. low = green, medium = blue, pink = high, the median is marked by the line that divides the boxes, the top and bottom of the box are the 75th and 25th percentiles respectively, and the whiskers show the minimum and maximum values.

There was no significant difference in the epibacterial abundance between the low and medium salinity treatments (GLM _negative binomial_, χ^2^_1_,_18_ = 27, *p* = 0.11, Figure 1A), but there was a significant increase in the abundance between T1 and T2 (GLM _negative binomial_, χ^2^_1_,_17_ = 22, *p* = 0.02) within these levels. In contrast, the high salinity treatment had significantly higher bacterial abundance than the low and medium treatment at T1 (GLM _negative binomial_, χ^2^_1_,_28_ = 44, *p* < 0.001) followed by a significant decrease at T2 (GLM _negative binomial_, χ^2^_1_,_8_ = 11, *p* = 0.02).

### Effect of Salinity and Time on Epibacterial Abundance and Richness

There was a significant overall effect of salinity on species richness (the number of observed taxa) at T1 (Kruskal–Wallis, χ^2^ = 9.9257, *p* = 0.006993, Table 1 and Figure 1B), but not T2 (Kruskal–Wallis, χ^2^ = 5.9154, *p* = 0.51). Specifically at T1 the high salinity treatment had significantly lower richness than the low salinity treatment (*Z* = −3.028, *p* = 0.007), however, no significant difference could be detected between high vs. medium salinity (*Z* = −2.19, *p* = 0.42) or medium vs. low salinity (Z = 0.96, *p* = 0.33) at T1.

**TABLE 1:** Kruskal–Wallis analysis of species richness between indicated treatment conditions.

Comparison	Species richness		
	χ^2^/z	DF	*p*-value
**Effect of salinity at T1**			
Global model	9.93	2	0.0070
High vs. Low	–3.028		0.0074
High vs. Medium	–2.19		0.43
Low vs. Medium	0.96		0.34
**Effect of salinity at T2**			
Global model	5.92	2	0.052
High vs. Low	NA		NA
High vs. Medium	NA		NA
Low vs. Medium	NA		NA
**Low salinity between time points**			
T0 vs. T1	4.5	1	0.039
T0 vs. T2	4.5	1	0.039
T1 vs. T2	6.0	1	0.014
**Medium salinity between time points**			
T0 vs. T1	5.0	1	0.025
T0 vs. T2	5.0	1	0.025
T1 vs. T2	6.8	1	0.009
**High salinity between time points**			
T0 vs. T1	5.0	1	0.025
T0 vs. T2	1.33	1	0.25
T1 vs. T2	3.75	1	0.053

In all treatments there was an initial increase in species richness (number of observed taxa) between T0 and T1, followed by a decrease between T1 and T2. The increase between T0 and T1 was significant in all treatments. However, the decrease between T1 and T2 was only significant in the low and medium treatments, but not the high salinity treatment (Figure 1B and Table 1). However, there were only two data points available for T2 at high salinity (see section Materials and Methods).

### Effect of Salinity and Time Point on Epibacterial Beta Diversity

**FIGURE 2:**
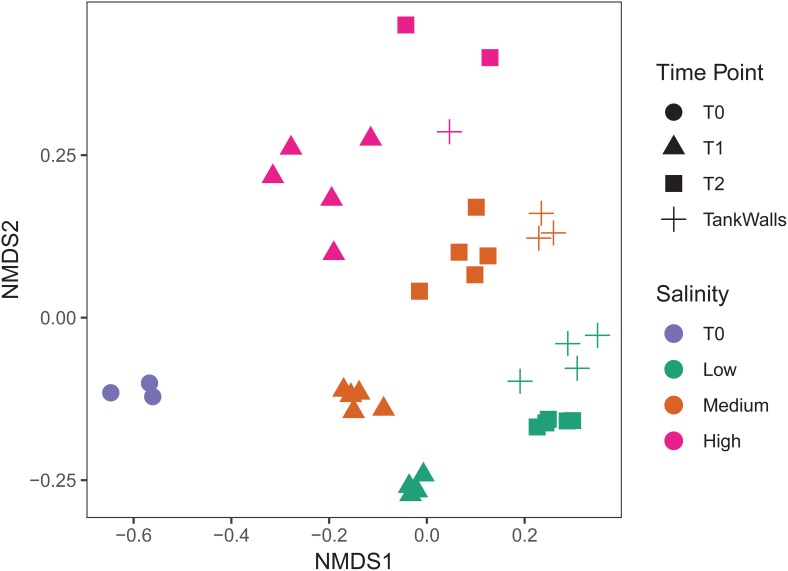
Epibacterial composition (NMDS based on Jaccard dissimilarity). Time point is indicated by shape (T0 = circles, T1 = triangles, T2 = squares), Salinity is indicated by color (low = green, medium = blue, pink = high). Tank walls are indicated by crosses (+).

There is clear differentiation between the salinity treatments and time points on the NMDS (Figure 2). Salinity had the largest explanatory power for community composition (PERMANOVA, *F*_2_,_25_ = 4.8, *p* = 0.001, and *R*^2^ = 0.26, Figure 2) followed by time point (PERMANOVA, *F*_1_,_25_ = 5.0, *p* = 0.001, and *R*^2^ = 0.14, Figure 2). The epibacterial communities on the tank walls were significantly different to the algal epibacterial communities (PERMANOVA, *F*_1_,_19_ = 2.4, *p* = 0.005, and *R*^2^ = 0.09). However, salinity had a greater influence than type of substrate (PERMANOVA, *F*_2_,_19_ = 4.3, *p* = 0.001, and *R*^2^ = 0.3).

### Effect of Salinity and Time Point on the Abundance of the Dominant Epibacterial Taxa

**FIGURE 3:**
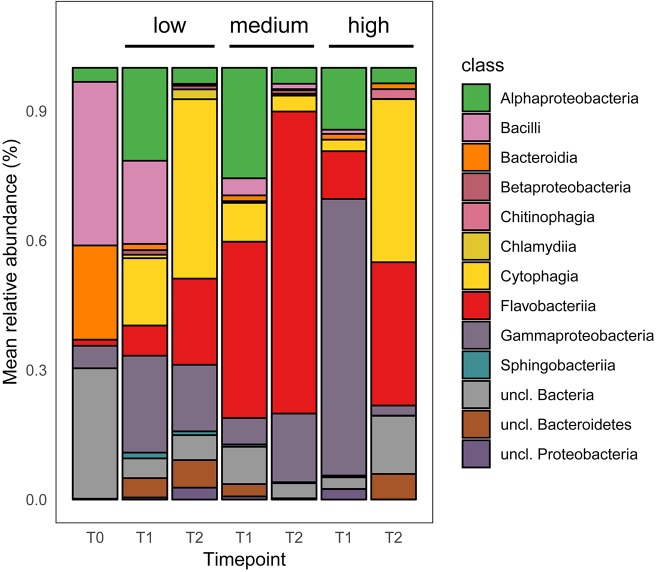
Mean relative abundance of top 200 dominant bacterial taxa at the class level [*n* = 5, excluding T0 (*n* = *3*), and T1 low salinity (*n* = 4)].

Differential abundance analysis of the contigs revealed a number of taxa whose abundance varied significantly between the salinity levels (Table 2 and Figure 3).

**TABLE 2:** Taxanomic assignments for contigs that varied significantly between pair-wise comparisons of indicated treatment conditions.

Phylum	Class	Genus species	Log2 Fold change	−/ +	*AdjP*-value
**Medium salinity vs. High salinity (+ = increase in medium salinity treatment)**					
Bacteroidetes	Chitinophagia	*Niabella ginsenosidivorans*	–21.5	–	3.2E-07
	Cytophagia	*Algoriphagus machipongonensis*	–23.0	–	8.7E-10
		*Echinicola vietnamensis*	4.2	+	2.0E-04
		*Marivirga tractuosa*	4.3	+	3.4E-05
		uncl. Cytophagales	–23.3	–	2.1E-08
		uncl. Cytophagales	4.1	+	4.9E-03
	Flavobacteriia	*Aequorivita sublithincola*	5.4	+	5.4E-07
		*Algibacter alginolytica*	8.0	+	4.1E-04
		*Chryseobacterium gallinarum*	–20.8	–	7.3E-07
		*Croceibacter atlanticus*	9.0	+	1.8E-03
		*Dokdonia* sp. 4H-3-7-5	8.8	+	8.5E-04
		*Dokdonia* sp. 4H-3-7-5	9.6	+	5.9E-03
		*Flavobacterium gilvum*	9.7	+	1.3E-03
		*Flavobacterium* sp. LPB0076	7.8	+	1.6E-05
		*Formosa agariphila*	9.5	+	7.7E-09
		*Formosa agariphila*	9.0	+	1.2E-04
		*Formosa agariphila*	9.7	+	6.3E-03
		*Formosa* sp. Hel3 A1 48	8.4	+	1.7E-03
		*Lacinutrix* sp. 5H-3-7-4	8.4	+	1.2E-03
		*Lacinutrix* sp. 5H-3-7-4	–5.8	–	5.9E-03
		*Lacinutrix* sp. 5H-3-7-4	9.0	+	6.3E-03
		*Lutibacter* sp. LP1	7.8	+	3.4E-06
		*Lutibacter* sp. LPB0138	9.4	+	8.2E-04
		*Lutibacter* sp. LPB0138	8.1	+	1.2E-03
		*Nonlabens dokdonensis*	9.3	+	1.8E-03
		*Polaribacter* sp. MED152	4.5	+	2.2E-04
		*Polaribacter vadi*	25.5	+	8.7E-10
		*Polaribacter vadi*	9.0	+	2.1E-03
		*Polaribacter vadi*	7.1	+	6.2E-03
		*Psychroflexus torquis*	–20.4	–	9.8E-07
		*Psychroflexus torquis*	9.7	+	1.6E-03
		*Tenacibaculum dicentrarchi*	9.8	+	1.1E-03
		*Tenacibaculum dicentrarchi*	8.7	+	5.9E-03
		uncl. *Dokdonia*	8.9	+	7.0E-04
		uncl. Flavobacteriaceae	–19.0	–	1.1E-06
		uncl. Flavobacteriaceae	–20.3	–	1.1E-06
		uncl. Flavobacteriaceae	8.3	+	2.9E-03
		uncl. Flavobacteriaceae	–6.3	–	5.9E-03
		*Weeksella virosa*	5.1	+	1.3E-06
		*Winogradskyella* sp. PG-2	–22.2	–	1.1E-07
		*Winogradskyella* sp. PG-2	10.7	+	1.5E-04
		*Winogradskyella* sp. PG-2	10.0	+	4.3E-04
Chlamydiae	Chlamydiia	*Simkania negevensis*	6.2	+	8.7E-10
Firmicutes	Bacilli	*Bacillus oceanisediminis*	–20.3	–	1.1E-06
		*Fictibacillus arsenicus*	6.5	+	1.3E-04
Proteobacteria	Alphaproteobacteria	*Hirschia baltica*	8.2	+	1.3E-04
		*Hirschia baltica*	8.4	+	4.3E-04
		*Kozakia baliensis*	6.4	+	2.4E-05
		Rickettsiales bacterium Ac37b	6.6	+	4.0E-03
	Gammaproteobacteria	*Morganella morganii*	5.3	+	1.2E-03
		*Pseudomonas moraviensis*	6.6	+	2.0E-03
		*Psychrobacter* sp. G	6.0	+	1.0E-05
		uncl. *Dickeya*	5.9	+	4.2E-04
		*Vibrio vulnificus*	–20.2	–	1.1E-06
uncl. Bacteria	uncl. Bacteria	uncl. Bacteria	6.7	+	6.5E-09
		uncl. Bacteria	–20.6	–	8.9E-07
**Low salinity vs. Medium salinity (+ = increase in low salinity treatment)**					
Bacteroidetes	Cytophagia	*Belliella baltica*	4.9	+	3.8E-06
		*Candidatus Amoebophilus asiaticus*	4.3	+	1.9E-03
		*Echinicola vietnamensis*	2.8	+	2.0E-03
		*Marivirga tractuosa*	–6.1	–	5.2E-06
		*Pontibacter korlensis*	5.0	+	5.2E-06
		*Pontibacter korlensis*	4.7	+	1.8E-03
		*Rufibacter* sp. DG31D	4.8	+	1.9E-04
		uncl. Cytophagales	4.7	+	2.0E-05
	Flavobacteriia	*Flavobacterium* sp. LPB0076	–5.1	–	3.6E-03
		*Formosa agariphila*	–6.9	–	6.7E-06
		*Formosa agariphila*	–6.4	–	4.1E-03
		*Lutibacter* sp. LP1	–4.9	–	2.7E-03
		*Maribacter* sp. 1 2014MBL MicDiv	–4.6	–	1.2E-03
		*Polaribacter vadi*	–37.8	–	7.6E-26
		*Tenacibaculum dicentrarchi*	–7.9	–	5.9E-03
		*Weeksella virosa*	2.4	–	3.6E-03
		*Winogradskyella* sp. PG-2	–7.3	–	7.2E-03
		*Winogradskyella* sp. PG-2	–7.2	–	8.6E-03
	uncl. Bacteroidetes	uncl. Bacteroidetes	5.0	+	7.8E-05
Proteobacteria	Alphaproteobacteria	*Hirschia baltica*	–6.1	–	4.0E-03
Proteobacteria	Gammaproteobacteria	*Enterobacter cloacae* complex sp. 35734	–4.9	–	2.7E-03
		*Legionella hackeliae*	–6.3	–	7.8E-06
		*Pseudomonas parafulva*	5.4	+	6.7E-06
		uncl. Alteromonas	–4.4	–	1.2E-03
		uncl. Gammaproteobacteria	–6.9	–	5.7E-03
	uncl. Proteobacteria	uncl. Proteobacteria	4.4	+	6.7E-06
uncl. Bacteria	uncl. Bacteria	uncl. Bacteria	–15.9	–	1.2E-06
		uncl. Bacteria	6.9	+	4.7E-05
		uncl. Bacteria	4.7	+	4.0E-03
**Low salinity vs. High salinity (+ = increase in low salinity treatment)**					
Bacteroidetes	Chitinophagia	*Niabella ginsenosidivorans*	–20.8	–	8.0E-07
	Cytophagia	*Algoriphagus machipongonensis*	–22.9	–	9.4E-10
		*Belliella baltica*	8.4	+	2.1E-12
		*Candidatus Amoebophilus asiaticus*	8.2	+	4.2E-08
		*Cyclobacterium amurskyense*	6.5	+	5.5E-03
		*Echinicola vietnamensis*	7.0	+	2.4E-11
		*Marivirga tractuosa*	6.3	+	1.4E-10
		*Marivirga tractuosa*	–4.3	–	3.4E-03
		*Pontibacter korlensis*	7.7	+	1.8E-10
		*Pontibacter korlensis*	7.5	+	1.9E-06
		*Rufibacter* sp. DG31D	7.7	+	2.6E-08
		uncl. Cytophagales	8.7	+	1.2E-11
		uncl. Cytophagales	–23.1	–	3.3E-08
	Flavobacteriia	*Aequorivita sublithincola*	6.4	+	1.7E-09
		*Chryseobacterium gallinarum*	–20.5	–	1.0E-06
		*Lacinutrix* sp. 5H-3-7-4	7.9	+	2.0E-03
		*Polaribacter* sp. MED152	6.7	+	6.7E-09
		*Polaribacter vadi*	–12.4	–	7.7E-03
		*Psychroflexus torquis*	–20.3	–	1.3E-06
		uncl. Flavobacteriaceae	–20.1	–	1.5E-06
		*Weeksella virosa*	7.4	+	7.1E-13
		*Winogradskyella* sp. PG-2	–21.6	–	2.7E-07
	uncl. Bacteroidetes	uncl. Bacteroidetes	7.4	+	7.0E-08
Chlamydiae	Chlamydiia	*Simkania negevensis*	7.2	+	7.1E-13
Firmicutes	Bacilli	*Bacillus oceanisediminis*	–20.1	–	1.5E-06
		*Fictibacillus arsenicus*	8.1	+	1.1E-06
Proteobacteria	Alphaproteobacteria	*Kozakia baliensis*	7.4	+	9.2E-07
	Gammaproteobacteria	*Pectobacterium* sp. SCC3193	7.6	+	4.7E-03
		*Pseudomonas parafulva*	8.3	+	3.6E-10
		*Psychrobacter* sp. G	6.9	+	5.6E-07
		uncl. *Dickeya*	7.4	+	8.0E-06
		*Vibrio vulnificus*	–20.5	–	1.0E-06
Proteobacteria	uncl. Proteobacteria	uncl. Proteobacteria	7.7	+	1.2E-11
uncl. Bacteria	uncl. Bacteria	uncl. Bacteria	–22.4	–	1.5E-11
		uncl. Bacteria	7.2	+	5.1E-10
		uncl. Bacteria	8.8	+	1.2E-06
		uncl. Bacteria	–20.4	–	1.2E-06
		uncl. Bacteria	6.9	+	5.3E-05
**Tank walls vs. Algae epibacteria (+ = increase on tank walls)**					
Bacteroidetes	Chitinophagia	*Saprospira grandis*	–2.8	–	2.6E-04
	Cytophagia	*Echinicola vietnamensis*	–2.5	–	8.6E-04
		*Marivirga tractuosa*	–1.9	–	3.4E-03
		*Marivirga tractuosa*	–1.9	–	6.3E-03
		*Pontibacter korlensis*	–2.3	–	1.5E-04
		uncl. Cyclobacteriaceae	–2.6	–	8.0E-03
	Flavobacteriia	*Algibacter alginolytica*	–4.3	–	3.2E-03
		*Chryseobacterium gallinarum*	–4.7	–	1.6E-06
		*Dokdonia* sp. 4H-3-7-5	–6.3	–	5.3E-04
		*Dokdonia* sp. 4H-3-7-5	–6.2	–	8.2E-04
		*Flavobacterium gilvum*	–6.2	–	8.3E-04
		*Flavobacterium* sp. LPB0076	–3.7	–	6.3E-03
		*Formosa agariphila*	–5.7	−	2.6E-04
		*Formosa agariphila*	–6.3	−	5.3E-04
		*Formosa* sp. Hel3 A1 48	–5.9	−	8.3E-04
		*Lacinutrix* sp. 5H-3-7-4	–6.3	−	8.3E-04
		*Lacinutrix* sp. 5H-3-7-4	–5.1	−	8.3E-03
		*Muricauda ruestringensis*	–2.4	−	2.6E-04
		*Non-labens dokdonensis*	–5.5	−	1.1E-03
		*Polaribacter* sp. MED152	–2.5	−	1.3E-03
		*Psychroflexus torquis*	–6.8	−	8.2E-04
		*Psychroflexus torquis*	–2.7	–	7.1E-03
		*Tenacibaculum dicentrarchi*	–5.2	–	4.4E-03
		uncl. Dokdonia	–5.8	–	8.6E-04
		uncl. Flavobacteriaceae	–5.1	–	1.1E-03
		*Weeksella virosa*	–2.2	–	1.7E-03
		*Winogradskyella* sp. PG-2	–5.6	–	6.5E-04
		*Winogradskyella* sp. PG-2	–5.2	–	4.3E-03
Proteobacteria	Alphaproteobacteria	*Bartonella henselae*	–4.0	–	4.4E-03
		*Bradyrhizobium diazoefficiens*	3.8	+	1.5E-04
		*Pelagibacterium halotolerans*	3.5	+	8.2E-04
		*Pelagibacterium halotolerans*	3.4	+	8.6E-04
		*Starkeya novella*	3.7	+	3.0E-04
		uncl. *Caulobacter*	4.0	+	1.5E-04
	Gammaproteobacteria	*Fluoribacter dumoffii*	3.5	+	2.1E-03
		*Legionella fallonii*	3.5	+	1.1E-03
		*Marinomonas* sp. MWYL1	3.5	+	8.6E-04
		uncl. Gammaproteobacteria	3.6	+	1.3E-03

#### Medium vs. High Salinity Treatment

Fifty-six taxa were found to be significantly differently abundant among the medium and high salinity treatments (Table 2). Thirteen species were significantly present in the high salinity treatment, while 43 species were significantly present in the medium salinity treatment. Irrespective of timepoints sampled, the bacterial taxon that on average was more prevalent at the high salinity was Cytophagia while Flavobacteriia, Gammaproteobacteria, and Alphaproteobacteria were significantly present in the medium salinity treatment when compared to the high salinity treatment (Figure 3). A number of Cytophagia species were significantly different in the high treatment, including *Algoriphagus machipongonensis, Belliella baltica, Candidatus Amoebophilus asiaticus* in contrast to the medium salinity. Other species like *Chryseobacterium gallinarum, Bacillus oceanisediminis, Vibrio vulnificus* were significantly abundant in the high salinity treatment. *Lacinutrix* sp. 5H-3-7-4, *Winogradskyella* sp. PG-2 and *Psychroflexus torquis* were present in both medium and high salinity treatment (Supplementary Figure S1 and Table 2).

#### Low vs. Medium Salinity Treatment

Twenty-nine taxa were found to be significantly different among the low and medium treatment. While Cytophagia significantly dominated the low treatment irrespective of timepoints, Flavobacteriia, unclassified Proteobacteria, unclassified Bacteria and Gammaproteobacteria were significantly more abundant on the medium salinity treatment (Figure 3). A number of Cytophagia species like *Belliella baltica, Candidatus Amoebophilus asiaticus, Echinicola vietnamensis, Pontibacter korlensis, Rufibacter* sp. DG31D, uncl. Cytophagales were significantly present in the low salinity treatment. Flavobacteriia species like *Formosa agariphila, Flavobacterium* sp. LPB0076, *Lutibacter* sp. LP1, *Polaribacter vadi, Tenacibaculum dicentrarchi, Weeksella virosa, Winogradskyella* sp. PG-2 were present in the medium salinity treatment. Among Gammaproteobacteria while *Pseudomonas parafulva* was present significantly in the low salinity treatment, *Enterobacter cloacae* complex sp. 35734 and unclassified Gammaproteobacteria were present in the medium treatment (Supplementary Figure S2 and Table 2).

#### Low vs. High Salinity Treatment

Thirty-eight taxa were found to be significantly different among the low and high salinity treatments, with Cytophagia, Chlamydiia, unclassified Proteobacteria and Gammaproteobacteria being significantly more abundant in the low salinity treatment while Flavobacteriia was equally well prevalent under both low and high salinity treatments (Figure 3). A number of Cytophagia species like *Belliella baltica, Candidatus Amoebophilus asiaticus, Cyclobacterium amurskyense, Echinicola vietnamensis, Pontibacter korlensis, Rufibacter* sp. DG31D were present in the low salinity treatment, while species like *Marivirga tractuosa*, unclassified *Cytophagales* were equally prevalent in both low and high salinity treatment. Among Flavobacteriia, *Aequorivita sublithincola, Lacinutrix* sp. 5H-3-7-4, *Polaribacter* sp. MED152, *Weeksella virosa* were present in the low salinity treatment while *Chryseobacterium gallinarum, Polaribacter vadi, Psychroflexus torquis* were present the high treatment (Supplementary Figure S3 and Table 2).

### Epibacterial Community From Tank Walls

Irrespective of the salinity level and timepoint, 38 taxa were found to be significantly different among tank walls and surface of *Agarophyton*. While Alphaproteobacteria and Gammaproteobacteria dominated the tank walls, Cytophagia, Flavobacteriia, and Chitinophagia were significantly more abundant on the epibacterial community of the seaweed (Supplementary Figure S4 and Table 2).

### ‘Core’ Epibacterial Taxa on *Agarophyton* Among All Salinity Levels and Timepoints

**FIGURE 4:**
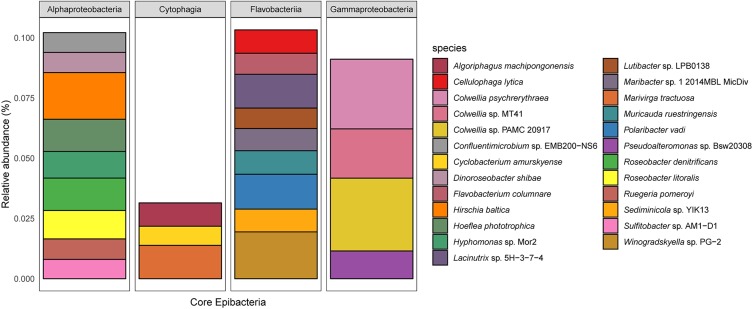
The relative abundance of the core epibacterial taxa that were shared between all salinity levels (top 25 most dominant shown, corresponding to 50% of the total abundance). The plot is faceted by Class and the color denotes the species. Shared taxa is defined as taxa present in all replicates for all treatments.

There were 737 taxa shared between all salinity levels and at all time points and were defined as ‘core’ epibacterial taxa. While 770 taxa were shared between medium and high salinity level, high and low salinity level shared 764 taxa. Highest level of taxa, i.e., 1332 were shared between medium and low salinity level. The most abundant taxa were Flavobacteriia, Cytophagia, Alpha and Gammaproteobacteria (Figure 4 and Supplementary Table S1).

## Discussion

In this study, we assessed the influence of three different salinities (low, medium, high) and time on the epibacterial abundance, richness and community composition of an invasive seaweed in a long term mesocosm experiment of 5 months duration. At both time points, i.e., T1 (August: 3 months after the start of the experiment) and T2 (October: end of the experiment, i.e., 5 months after the start of the experiment) (i) salinity was found to be a significant factor determining epibacterial abundance, richness and community composition of *Agarophyton* between high and low salinity level and (ii) time played a significant role in altering species richness and community composition between T1 and T2 at all salinity levels. We observed a highly diverse community of algae associated bacteria with 120697 contigs assigned to 2538 bacterial species. Variability of the bacterial microbiome between replicate algal individuals was not significantly high in comparison to the between treatment variation, as indicated by the tightly formed clusters (Figure 2).

The salinity level tested in the current study, i.e., 8.5, 16.5, and 25.5 psu are comparable to those tested by Stratil et al. (2014), i.e., 5, 19, and 25 psu on the epibacterial communities of the brown seaweed *Fucus vesiculosus*. Following a 14d treatment Alphaproteobacteria, Gammaproteobacteria, Bacteroidetes and Betaproteobacteria were more abundant on the *Fucus* surface at 5 psu. Alphaproteobacteria, Gammaproteobacteria and Bacteroidetes dominated the surface of *Fucus* at 19 and 25 psu. However, in our experiment Cytophagia, Bacilli, Alphaproteobacteria and Gammaproteobacteria dominated in low salinity (8.5 psu) at T1 and Cytophagia, Flavobacteriia and Gammaproteobacteria dominated at T2. In the current study Flavobacteriia, Gammaproteobacteria, Alphaproteobacteria and Cytophagia dominated the surface of *Agarophyton* at medium (16.5 psu) in T1 after 3 months, while Flavobacteriia and Gammaproteobacteria dominated at T2 after 5 months of experiment. For the high treatment (25.5 psu), Gammaproteobacteria, Flavobacteriia and Alphaproteobacteria dominated in T1 while Cytophagia, Flavobacteriia and unclassified bacteria dominated at T2. The observed differences between our study and Stratil et al. (2014) may be explained by the fact that epibacterial communities are known to differ among different genera of seaweeds (Lachnit et al., 2009). Moreover, the duration of these two experiments was not similar (14 days vs. 5 months) and this time difference may also have resulted in differences among the bacterial groups colonizing the surfaces of *Fucus* and *Agarophyton*. For example, season is known to influence the bacterial community composition of seaweeds under field conditions (Lachnit et al., 2011). Similar to the observation made by Lachnit et al. (2011) with *Agarophyton* (collected freshly from Kiel fjord with average salinity 16 psu) in July 2007 and July 2008, Alphaproteobacteria and Bacteroidetes dominated the surface of *Agarophyton* in our T1 samples (i.e., August) but the community was dominated by Flavobacteriia and Gammaproteobacteria at T2 (i.e., October) at the medium salinity treatment, i.e., 16.5 psu. These findings confirm that seaweeds harbor temporally adapted epibacterial biofilms on their surfaces (Lachnit et al., 2011).

Cytophagia belongs to the group Bacteroidetes, which often have agarolytic and carrageenanolytic properties (Martin et al., 2014). At T2, Cytophagia dominated the high salinity level (Figure 3). Although the seaweeds were harvested from a site in the North Sea that is exposed to relatively high salinities of up to 35 psu, the high salinity treatment samples did not perform very well between T1 to T2 under laboratory conditions. This was indicated by a significant gradual decline in biomass between T1 and T2 when compared to low and medium salinity samples (data not shown). Three out of five replicates from the high salinity treatment could not be harvested due to fragile and decayed thallus conditions for much of the algal material.

Noticeably, bacterial abundance at high salinity was significantly higher at T1, where Gammaproteobacteria and Alphaproteobacteria dominated the algal surface. *Vibrio vulnificus*, an ubiquitous toxigenic bacterium found in coastal environments (Mahmud et al., 2008) and a human pathogen was highly abundant in the high salinity treatment and may have contributed to the degradation and breakdown of algae at the high salinity treatment. Gonzalez et al. (2014) found that *Vibrio vulnificus* is naturally associated with natural populations of *Agarophyton* in both summer and early fall in mid-Atlantic coast region. Decreased species richness in high salinity as observed in our study was in accordance to the observation made by Pavloudi et al. (2017) where low bacterial diversity was found in marine sediment bacteria at high salinity level but contradicted Remane’s species minimum concept.

A higher abundance of chitin degrading bacteria like *Niabella ginsenosidivorans* belonging to Chitinophagia in the high salinity treatment may indicate an attraction to the degraded cell wall components of the algae and thereby utilization of the degraded cell wall products (Michel et al., 2006). Moreover, certain chitin degrading bacteria like *Chitinophaga salinosoli* are known to be highly salt tolerant (Dong et al., 2018) which may explain abundance of this group in the high salinity level.

While certain species like *Echinocola vietnamensis, Pontibacter korlensis, Marivirga tractuosa* belonging to Cytophagia dominated both medium and low salinity levels; certain bacteria like *Algoriphagus machipongonensis* belonging to Cytophagia was significantly present in the high salinity treatment. Flavobacteriia is a very diverse group and found in many different environments and in/on different host organisms (Lachnit et al., 2011; Jackson et al., 2012; Schmitt et al., 2012). *Polaribacter vadi, Winogradskyella* sp. PG-2, *Formosa agariphila, Polaribacter* sp. MED152, *Tenacibaculum dicentrarchi, Lacinutrix* sp. 5H-3-7-4, and *Psychroflexus torquis* were highly abundant in the medium salinity level (i.e., average salinity of Baltic Sea) compared to low and high level. Marine Bacteroidetes, in general, and marine Flavobacteriia, in particular, have been described to be degraders of biopolymers, such as proteins and polysaccharides (for example, Thomas et al., 2011). This view is supported by high Flavobacteriia abundances in nutrient-rich habitats, such as aggregates of particulate organic matter (Williams et al., 2013) or in the microbiota of marine algae and invertebrates (Di Camillo et al., 2012; Dong et al., 2012). Algal polysaccharide degrading bacteria *Lacinutrix* sp. 5H-3-7-4, *Formosa* agariphila and the halophilic bacteria *Psychroflexus torquis* were present in both medium and high salinity treatments. *Pontibacter korlensis* is known to have anti-microbial and anti-biofilm activities (Balan et al., 2016) and may offer associational defense to the alga as is known from other seaweed associated bacteria. In general Cytophagia, Flavobacteriia, Gammaproteobacteria, and Chitinophagia dominated the algal surface at T2, with Alphaproteobacteria being more abundant on the tank walls. These bacterial taxa therefore can be considered the ‘core’ epibacterial taxa. This may be explained by the observation that bacteria belonging to CFB *group* are known to be associated with seaweeds in general at a higher percentage compared to Alphaproteobacteria (reviewed by Hollants et al., 2013).

There are two possible explanations for the observed shifts in epibacterial richness and diversity in response to salinity: (1) direct physiological changes of the host alga and (2) via altered biotic interactions. The former can cause resulting alterations of exuded carbon and changes in the surface chemistry of the alga affecting epibacteria (Sudatti et al., 2011; Saha et al., 2014). Seaweed metabolites are known to affect bacterial attachment and growth (reviewed by Saha et al., 2017). Also, it has been demonstrated that seaweed surface metabolites can influence the overall epibacterial community composition when compared to non-living substrata (Lachnit et al., 2010) and can selectively attract beneficial and repel pathogenic bacteria (Saha and Weinberger, 2019). Thus, only microorganisms which can adapt to the specific surface chemistry conditions on a host surface might be able to settle on it, fitting the ‘niche’ model (Dumbrell et al., 2010). Altered biotic interactions may include metabolic competition or co-operation (Elias and Banin, 2012) among members of the epibacterial community. While grazers (micro and macro) and macrofoulers may generally also alter biotic interactions we can rule out their effect in the context of our experiment as they were excluded from the setup.

In temperate regions the bacterial community composition in the water column and the settling pressure of colonizing organisms undergoes strong seasonal shifts (Schauer et al., 2003). Thus, we may speculate that a potential change in the bacterioplankton community could have resulted in change in the microbiome on the surface of *Agarophyton* between different timepoints. To cope with seasonally fluctuating colonizing pressure, macrophytes including seaweeds in turn have been reported to seasonally up or downregulate the production of antifouling defense (Saha and Wahl, 2013; Wang et al., 2017; Guan et al., 2019) influencing seaweed-bacteria interaction and also interaction with other foulers. We have recently demonstrated that the surface chemistry of *Agarophyton* is capable of selectively recruiting bacterial colonizers (Saha and Weinberger, 2019) and from previous studies we are aware that the antifouling defense of seaweeds including *Agarophyton* undergoes seasonal fluctuations indicating a change in the surface chemistry of the alga over time (Saha and Wahl, 2013; Wang et al., 2018). Although in this current study we have not investigated the surface metabolome of *Agarophyton* at different timepoints, i.e., T0, T1, and T2, we may speculate that a seasonal fluctuation in the surface chemistry of the algae may have resulted in the selective recruitment of the bacterial colonizers on the surface of the alga and thereby different community composition at different timepoints.

Extensive 16S rRNA gene sequencing of the bacterial community of *Ulva australis* only detected 6 bacterial species of a total of 528 being common between six individual algae (Burke et al., 2011) and the same approach only detected 10 bacterial species of a total of 4341 being common among 25 individual specimens of *Fucus vesiculosus* (Stratil et al., 2013). Irrespective of sampling timepoint and salinity level, we could demonstrate for the first time the existence of ‘core’ microbial taxa (upto the species level) on seaweed surfaces and detected much higher number of species, i.e., 737 which were consistently found on the surface of *Agarophyton* and was dominated by Flavobacteriia, Cytophagia, Alpha and Gammaproteobacteria. This could be a result of different sequencing techniques used in the current study.

## Conclusion

We can state that salinity and time can shape epibacterial community composition of seaweeds. The observed compositional differences due to salinity were attributed to a few members of the community like Gammaproteobacteria, Flavobacteriia and Cytophagia. However, individual species varied significantly among the treatments and several species also formed the ‘core’ bacterial microbiome irrespective of treatment level and timepoint. We do not know yet the exact mechanisms driving these shifts and factors responsible for maintaining ‘core’ bacterial species. By comparing the end time point data of the algal epibacteria with non-living reference substrata (Supplementary Figure S4), we showed that salinity has a direct influence in shaping epibacterial richness and community on algal surface. We are currently unable to distinguish the factors in such a complex and dynamic holobiont system.

So far most manipulative experiments have been conducted for short periods only, ignoring the natural biogenic and seasonal fluctuations in the water column (Wahl et al., 2016). We found significant shifts in abundance, richness, and community composition between T1 and T2, indicating that long term experiments incorporating biogenic and seasonal fluctuations are required to fully understand epibacterial succession over time under the influence of abiotic stressors. Given the established role of epibacteria in modulating the interaction of the algal hosts with its environment, we predict that the complex shifts brought by salinity may change these interactions influencing health and fitness of the host. However, this has not been tested in the current study and deserves future investigations. It has been recently demonstrated that bacterial communities can help the native filamentous brown seaweed *Ectocarpus* to adapt to salinity gradients (Dittami et al., 2016), facilitating transition from marine to freshwater medium. Root microbial communities can also indirectly control the success of invasive macrophytes in marine ecosystems (Gribben et al., 2017). We do not know whether salinity induced change in epibacterial community composition and maintenance of a set of ‘core’ bacterial species despite salinity stressors as observed in the current study may aid invasion success of seaweeds and thus deserves future investigation. Also, it will be interesting to investigate how such a shift in epibacterial communities (as demonstrated in our study) may or may not alter the functions provided by these bacteria and may indirectly control the success of invasive seaweeds in the marine ecosystems.

## Data Availability Statement

The datasets generated for this study can be found in the PANGEAE repository (Saha, 2019). The R scripts detailing the analysis can be found on Github: https://github.com/rmwferguson/algalMetagenomicAnalysis. Raw sequence data have been submitted to the European Nucleotide Archive under project accession PRJEB34406 (samples ERS3759191–ERS3759242).

## Author Contributions

MS designed the study, contributed conceptually, performed the experiments, and wrote the manuscript. RF analyzed the data, produced plots, and assisted in writing the manuscript. SD assisted in running the experiments and subsequent samplings. RM generated the data on bacterial densities. FP helped with sampling at different stages of the experiments. SK did the sequencing work while SN did the bioinformatics. FW provided conceptual contributions to the experiments. All authors reviewed the manuscript.

## Conflict of Interest

SN is co-founder of omics2view.consulting GbR. The remaining authors declare that the research was conducted in the absence of any commercial or financial relationships that could be construed as a potential conflict of interest.

## Supplementary Material

FIGURE S1 Taxonomic assignments and log2 fold change for contigs that varied significantly between the high and medium salinity treatments (*n* = 5, Wald test, *p*-values were adjusted with the Benjamini–Hochberg method).

FIGURE S2 Taxonomic assignments and log2 fold change for contigs that varied significantly between the medium and low salinity treatments (*n* = 5, Wald test, *p*-values were adjusted with the Benjamini–Hochberg method).

FIGURE S3 Taxonomic assignments and log2 fold change for contigs that varied significantly between the high and low salinity treatments (*n* = 5, Wald test, *p*-values were adjusted with the Benjamini–Hochberg method).

FIGURE S4 Taxonomic assignments and log2 fold change for contigs that varied significantly between the epibacterial and tank wall communities (*n* = 3, Wald test, *p*-values were adjusted with the Benjamini–Hochberg method).

TABLE S1 Core epibacteria (bacterial taxa found on all replicates at all salinity levels) sorted in order of total number of sequences.
