# Supplementary figures and images for: Salinity and Time Can Alter Epibacterial Communities of an Invasive Seaweed
Source: Front Microbiol. 2020 Jan 15;10:2870. doi: 10.3389/fmicb.2019.02870 (PMC6974479; doi:10.3389/fmicb.2019.02870)

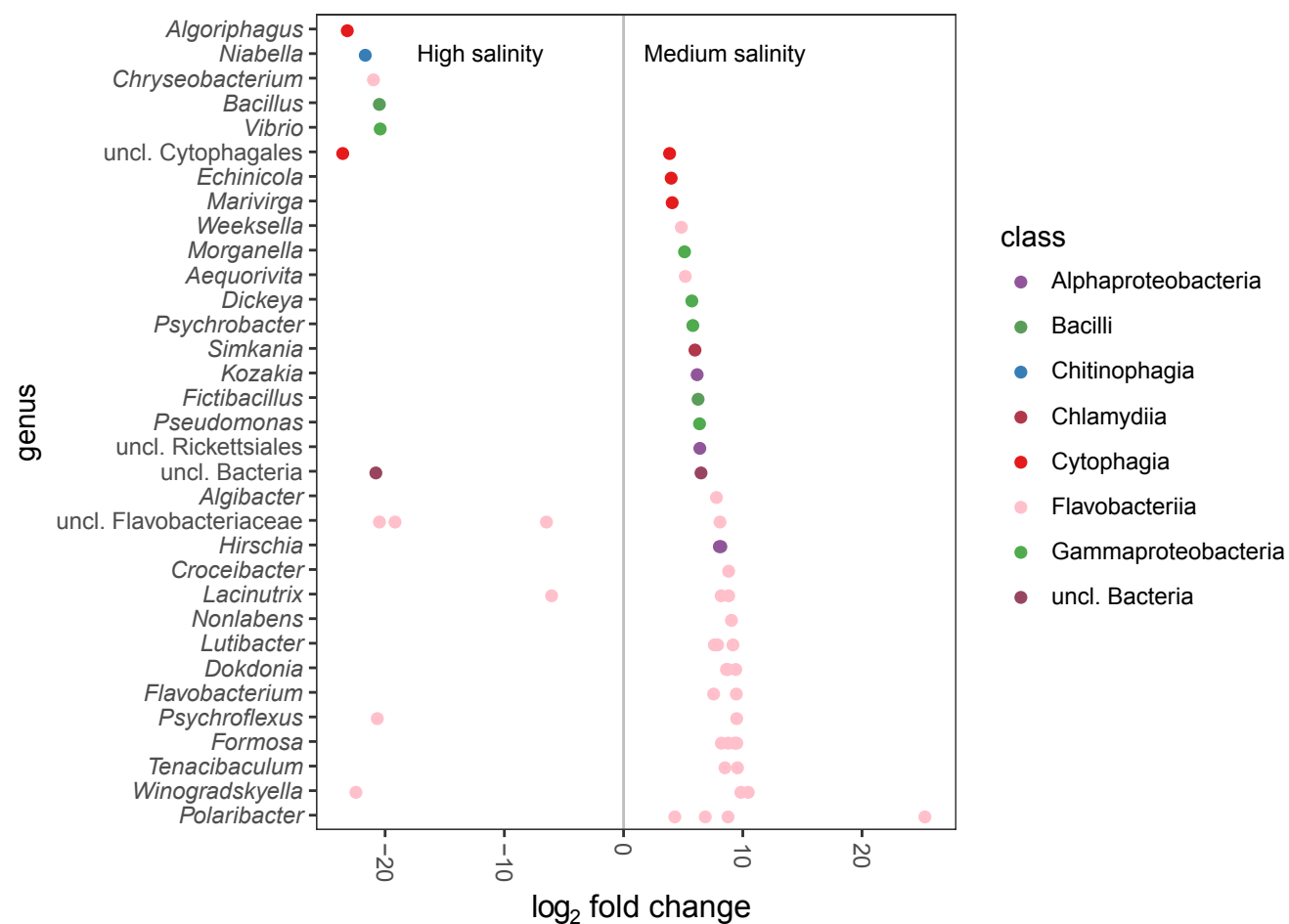

Supplement: FIGURE S1 — Taxonomic assignments and log2 fold change for contigs that varied significantly between the high and medium salinity treatments (n = 5, Wald test, p-values were adjusted with the Benjamini–Hochberg method). [file Image_1.pdf]

genus

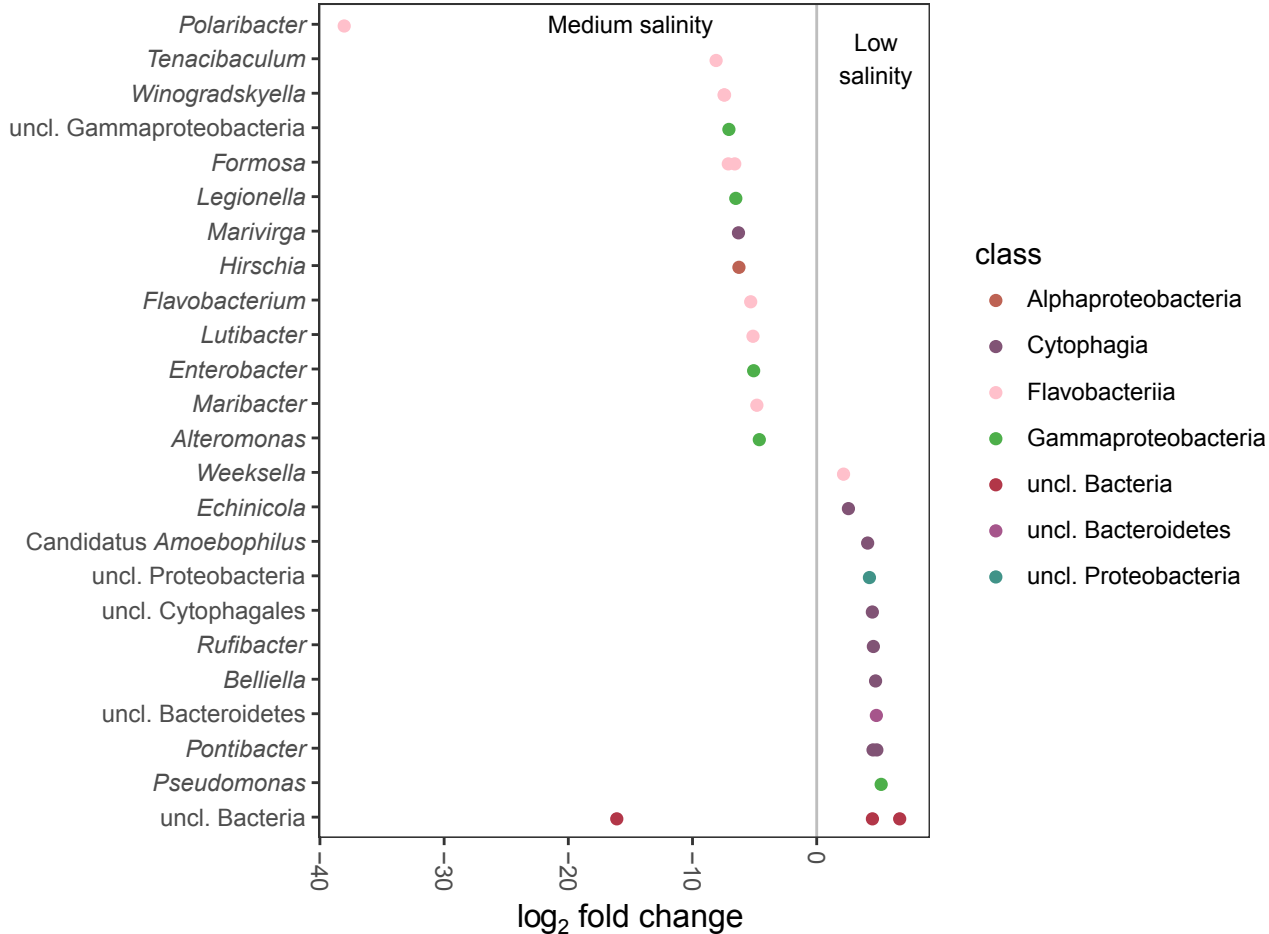

Supplement: FIGURE S2 — Taxonomic assignments and log2 fold change for contigs that varied significantly between the medium and low salinity treatments (n = 5, Wald test, p-values were adjusted with the Benjamini–Hochberg method). [file Image_2.pdf]

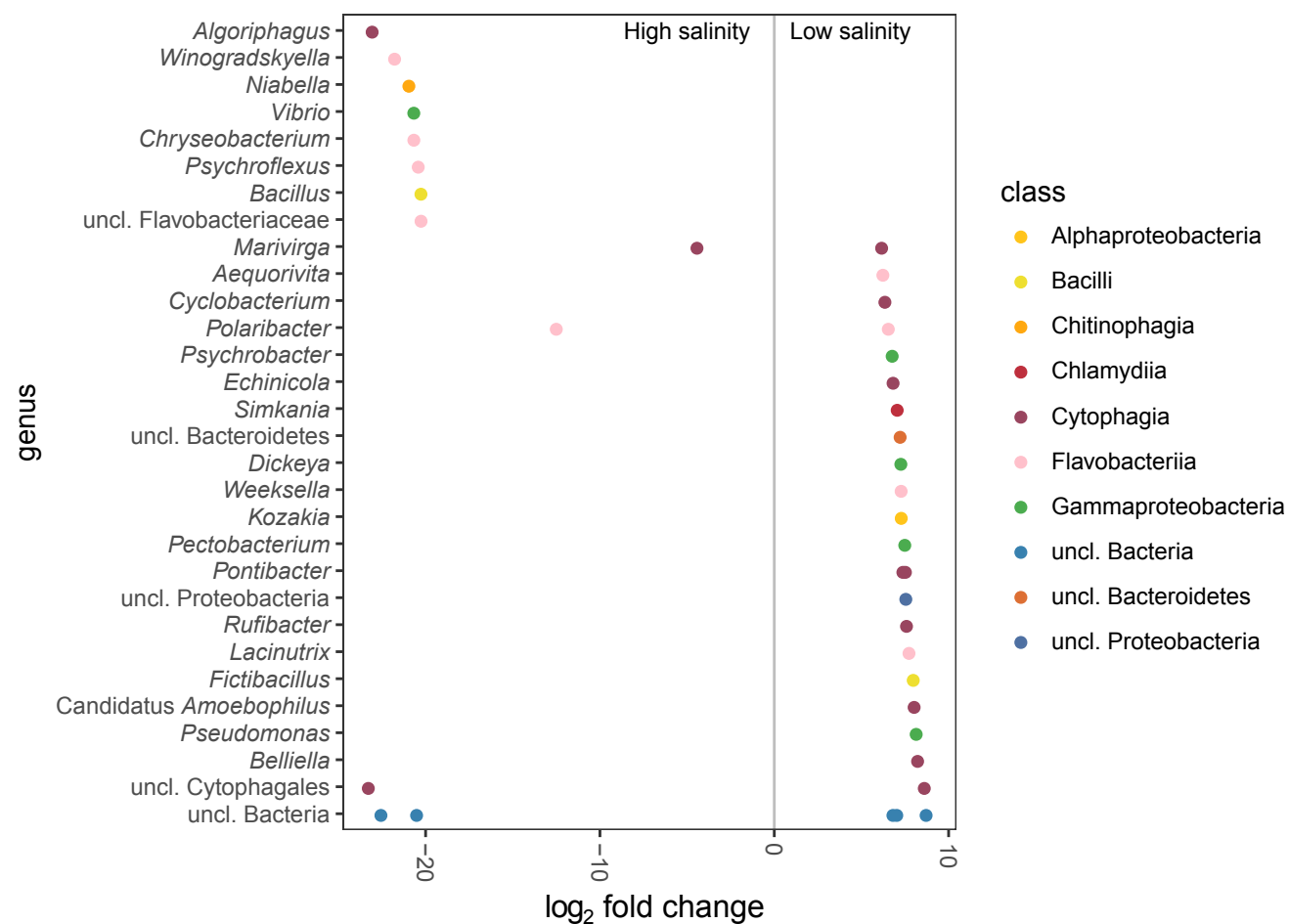

Supplement: FIGURE S3 — Taxonomic assignments and log2 fold change for contigs that varied significantly between the high and low salinity treatments (n = 5, Wald test, p-values were adjusted with the Benjamini–Hochberg method). [file Image_3.pdf]

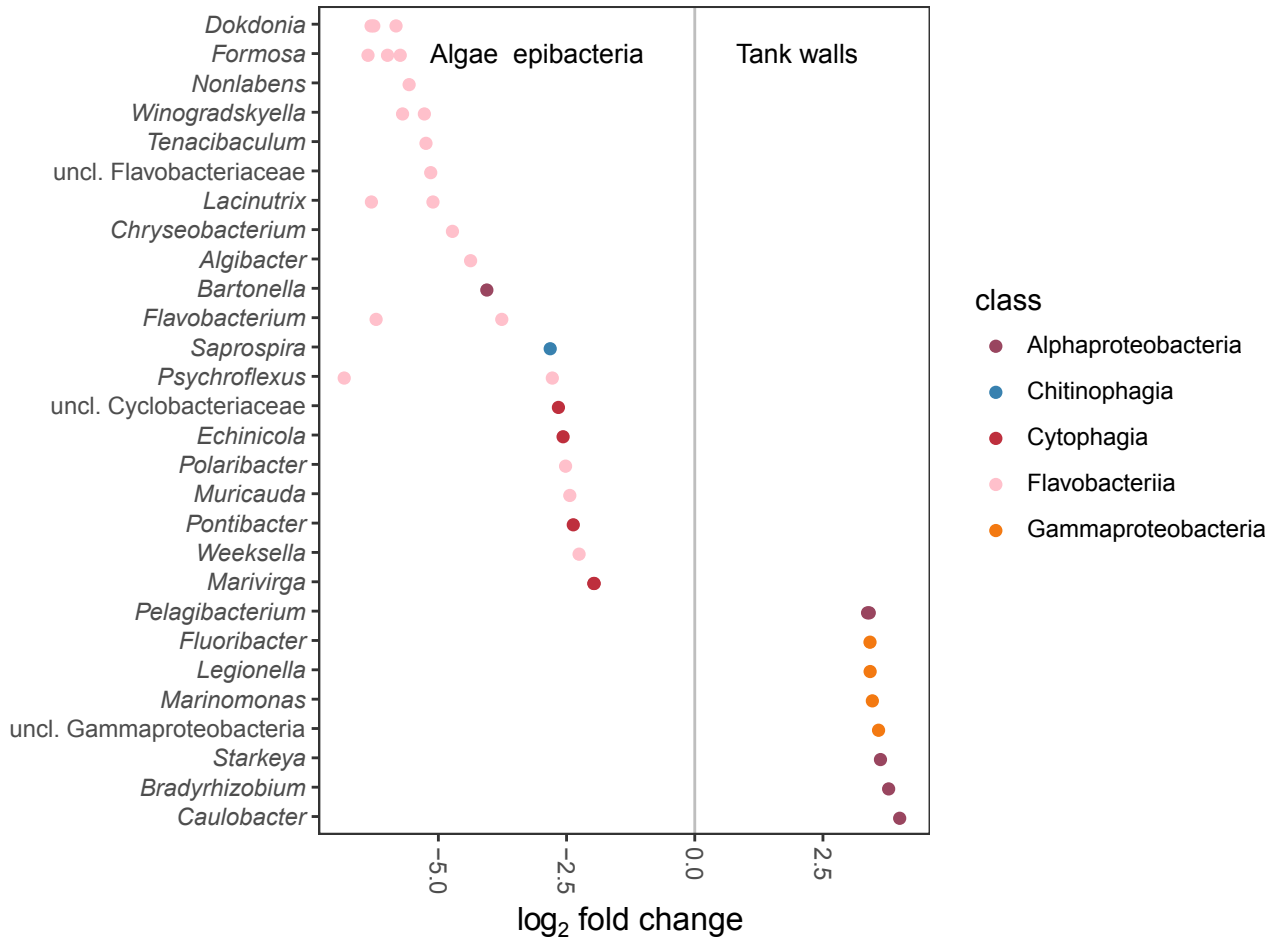

Supplement: FIGURE S4 — Taxonomic assignments and log2 fold change for contigs that varied significantly between the epibacterial and tank wall communities (n = 3, Wald test, p-values were adjusted with the Benjamini–Hochberg method). [file Image_4.pdf]

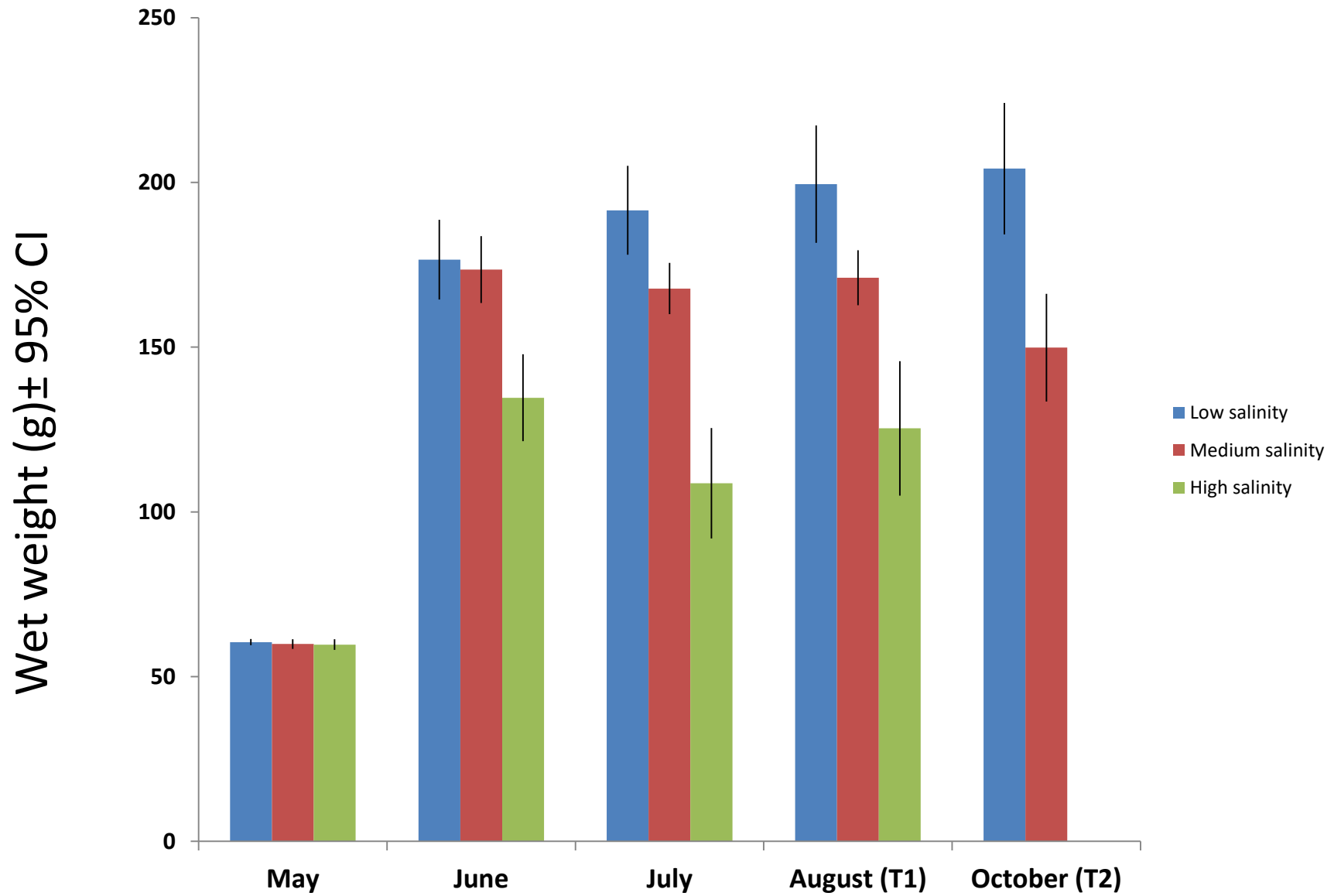

Supplement: Supplementary file 7 [file Data_Sheet_1.PDF]
