# Supplementary material for: Salinity and Time Can Alter Epibacterial Communities of an Invasive Seaweed
Source: Front Microbiol. 2020 Jan 15;10:2870. doi: 10.3389/fmicb.2019.02870 (PMC6974479; doi:10.3389/fmicb.2019.02870)
Supplement: Supplementary file 6 [file Data_Sheet_2.docx]

**Appendix S1**

**Quantification of epibacteria associated with the surface of *Agarophyton***

In order to assess the bacterial cell density on *Agarophyton*, 140mg of young algal tips were sonicated with 20 sterile glass beads and 7.5ml of artificial seawater (of salinities of respective treatments) at 20^o^C, vortexed for 3min, followed by decantation of the water into polycarbonate filters (0.2µm, Ø = 25mm Black, DHI Denmark). The algal material was then shaken vigorously with 7.5ml of artificial seawater and the water was decanted. This step was repeated two more times. Bacteria on the surface of the filter (using only a quarter of the filter) were investigated by epiﬂuorescence microscopy using a maginification of 630x (Axio Imager.Z1, Zeiss, Jena, Germany). Prior to counting the filters were stained with 0.2% (v/v) 4,6 - diamidino-2-phenylindole (DAPI, Life Technologies GmbH, Darmstadt, Germany). With the help of the computer program ProgRes CapturePro, five pictures were taken of five random fields per filter. On each picture 20 randomly scattered squares of 50µm^2^ were generated by help of in image analysis software, Image J (plugin “ROI manager”).
